# Tibial morphological variations reveal medial proximal tibial angle discrepancies in Japanese patients: Three‐type classification for high tibial osteotomy

**DOI:** 10.1002/jeo2.70511

**Published:** 2025-11-05

**Authors:** Etsuko Tabata, Ryuichi Nakamura, Hiromi Yamaguchi, Ryosuke Asa, Masaki Takahashi, Kazunari Kuroda, Yasuo Katsuki

**Affiliations:** ^1^ Department of Radiological Technology Kanazawa Munehiro Hospital Kanazawa Japan; ^2^ Department of Radiological Technology Yawata Medical Center Komatsu Japan; ^3^ Joint Preservation and Sports Orthopaedic Center, Harue Hospital Sakai Japan; ^4^ Japan Advanced Institute of Science and Technology Nomi Japan; ^5^ Department of Orthopaedic Surgery Yawata Medical Center Komatsu Japan

**Keywords:** alignment, around‐knee osteotomy, knee osteoarthritis, tibial morphology, varus deformity

## Abstract

**Purpose:**

This study aimed to characterize the tibial morphology among Japanese patients with varus knee osteoarthritis and examine whether the mechanical and proximal anatomical medial proximal tibial angles (mMPTA and paMPTA) are equivalent in high tibial osteotomy (HTO) planning.

**Methods:**

In this retrospective study, 193 varus‐aligned knees (hip–knee–ankle [HKA] angle < 0°) from 209 Japanese patients undergoing total knee arthroplasty or HTO were analyzed. Full‐length lower limb radiographs were used to assess tibial morphology, with the tibia divided into proximal, middle, and distal segments. Based on the proximal–middle and middle–distal angular relationships, nine morphological subtypes were identified and subsequently grouped into three clinically relevant categories: medial bowing, straight, and lateral bowing. The discrepancy between mMPTA and paMPTA (ΔMPTA) was calculated for each morphological category. Data were analyzed using Kruskal–Wallis test, chi‐square test, paired *t*‐test, and ANOVA.

**Results:**

Tibial deformities were highly prevalent, with proximal and distal deformities observed in 67.9% and 50.8% of knees, respectively. Among the 193 knees, 49 (25.4%), 111 (57.5%) and 33 (17.1%) were classified as medial bowing, straight type, and lateral bowing types, respectively. Significant differences in ΔMPTA were observed among the three groups (*p* < 0.001). Medial bowing type showed a significant negative ΔMPTA ( − 1.5° ± 0.5°, *p* < 0.001), straight type showed negligible discrepancy (0.0° ± 0.7°, *p* = 0.60), and lateral bowing type demonstrated a significant positive ΔMPTA ( + 1.3° ± 1.0°, *p* < 0.001).

**Conclusions:**

Japanese patients with varus knee osteoarthritis exhibit complex tibial morphologies involving both proximal and distal segments. These morphological variations significantly influence the relationship between mMPTA and paMPTA. In 42.5% cases (non‐straight types), conventional equivalence assumptions may lead to intraoperative measurement errors during HTO verification. Preoperative morphological type identification and corresponding intraoperative adjustments based on predictable ΔMPTA values may enhance surgical accuracy and clinical outcomes.

**Level of Evidence:**

Level III.

**Abbreviations**
AKO: around‐knee osteotomies
BMD: bone mineral density
BMI: body mass index
dAA: distal anatomical axis
HKA: hip‐knee‐ankle
HTO: high tibial osteotomy
ICC: intraclass correlation coefficient
K–L: Kellgren–Lawrence
mAA: middle anatomical axis
MD: middle‐distal
mMPTA: mechanical medial proximal tibial angle
OA: osteoarthritis
pAA: proximal anatomical axis
paMPTA: proximal anatomical medial proximal tibial angle
PM: proximal‐middle
TKA: total knee arthroplasty
ΔMPTA: difference between mMPTA and aMPTA

## INTRODUCTION

Around‐knee osteotomies have become established for managing medial compartment osteoarthritis [26, 33]. Accurate deformity analysis is crucial for preoperative evaluation. Optimal outcomes depend on precise assessment and surgical technique [2, 3, 15, 21, 26, 27, 30].

Deformity evaluation using full‐length radiographs has been standard practice [5, 11, 28, 36]. Paley′s terminology has become the gold standard in deformity analysis [28]. He defined the mechanical axis as a straight line connecting the centers of adjacent joints and the anatomical axis as a line traversing the mid‐diaphyseal region of a bone.

For the tibia, the medial proximal tibial angle (MPTA) can be measured using mechanical (mMPTA) or anatomical (aMPTA) axes, defined as the angle between the respective axis and the tibial plateau [27]. Because the mechanical and anatomical axes of the tibia are generally considered to run parallel, mMPTA and aMPTA have traditionally been assumed to be equivalent. Accordingly, prefixes have often been omitted in clinical descriptions.

In clinical settings, mMPTA is used for surgical planning because full‐length radiographs depict the total tibial length. Conversely, aMPTA is used intraoperatively, as fluoroscopy offers limited field of view, encompassing only the proximal third of the tibia. This introduces potential inconsistencies between preoperative mechanical axis planning and intraoperative fluoroscopic verification. While wedge angle calculations are based on full‐length mechanical axis measurements, intraoperative confirmation of correction relies on limited‐view assessment, potentially leading to systematic measurement discrepancies.

Previous studies have reported that tibial morphology in Asian populations, particularly Japanese individuals, differs significantly from that of Caucasians [8, 23, 24, 32, 35, 38]. While European studies have demonstrated gender‐specific differences in knee morphology [12, 34], Japanese populations show distinct constitutional characteristics. Japanese patients have smaller MPTA values and a higher prevalence of varus deformity [8]. Various anatomical variations in tibial curvature have been documented, including combined deformities involving both proximal and distal segments of the tibia [23].

However, a critical question arises in clinical practice: whether the anatomical MPTA measured intraoperatively should be used interchangeably with the mechanical MPTA used in preoperative planning. This concern is particularly relevant for the proximal anatomical MPTA (paMPTA) measured from the limited tibial segment visible under intraoperative fluoroscopy, which differs from Paley′s classical definition but represents the practical measurement available during surgery. While discrepancies between preoperative mechanical and intraoperative proximal anatomical measurements could lead to surgical errors, no study has systematically investigated paMPTA as an independent parameter.

In clinical practice, numerous cases with complex deformities involving both ends of the tibia have been observed, rather than a single, straight axis. Additionally, discrepancies between preoperative mMPTA and intraoperative paMPTA have been observed, despite the common assumption that these values are equivalent. Although most studies have focused on proximal tibial deformities, few have addressed distal deformities, and detailed characterizations of tibial curvature patterns are lacking. Importantly, no study has directly examined the relationship between mMPTA and paMPTA, particularly in Japanese populations [23].

Discrepancies between mMPTA and paMPTA could lead to misjudgments in correction magnitude during high tibial osteotomy (HTO), where accurate angle measurements are critical for optimal alignment. Therefore, the aims of this study were to (1) analyze tibial morphology in Japanese patients with varus knee OA, (2) systematically quantify the relationship between mMPTA and paMPTA, and (3) provide morphology‐specific correction factors for clinical application in HTO. The hypothesis was that multiple deformity locations contribute to significant differences between these two angular measurements.

## MATERIALS AND METHODS

### Study design and patient selection

This retrospective study was approved by the institutional review board of the hospital (Approval No. 2021‐11). Given the retrospective nature of this study using anonymized radiographic data, the requirement for individual informed consent was waived by the ethics committee. A total of 209 patients (224 knees) presenting with varus alignment—defined as a hip–knee–ankle (HKA) angle of <0°—were included. These patients underwent either total knee arthroplasty (TKA) or HTO for the treatment of medial compartment knee OA and/or osteonecrosis between November 2019 and December 2020. Exclusion criteria were post‐traumatic OA, history of HTO, previous TKA or total hip arthroplasty on the affected side, and knees with severe deformities characterized by a flexion contracture > 20°, which significantly impair the accuracy of radiographic and mechanical axis measurements [7, 18].

The cohort included patients undergoing both TKA and HTO based on consecutive enrollment of patients with available full‐length lower limb radiographs, ensuring unbiased case selection within this surgical population without selection based on morphological features. The choice between TKA and HTO was made at the discretion of the treating surgeon, based on clinical factors such as patient age, activity level, and OA grading.

The following patient data were collected: age, sex, body mass index (BMI), bone mineral density (BMD) measured at the lumbar spine using dual‐energy X‐ray absorptiometry (DEXA), HKA angle, and Kellgren–Lawrence (K–L) grade of OA.

### Radiographic measurements and analysis

All radiographic measurements were independently performed by the first and fourth authors, who were blinded to the clinical details and treatment modalities. Measurements were conducted at separate times, and each observer was unaware of the other's findings.

**Figure 1:**
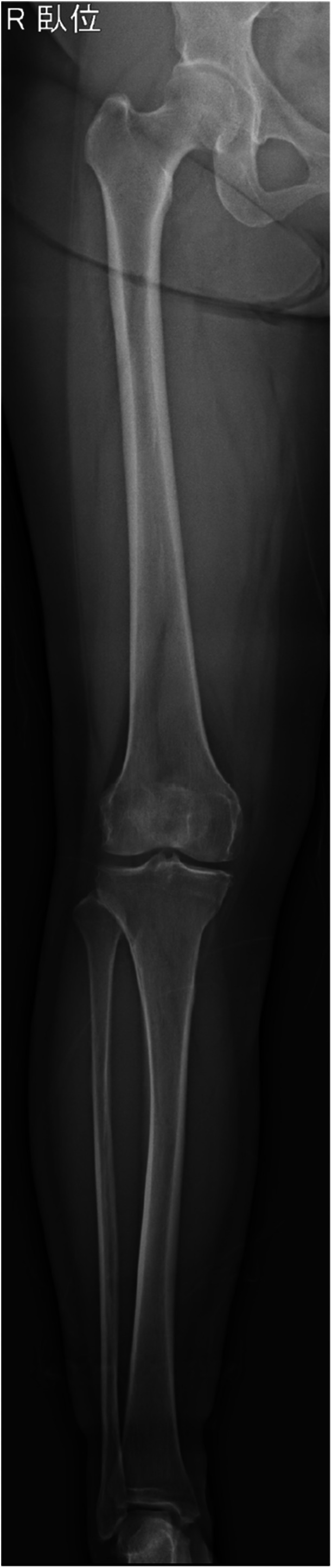
Representative full‐length lower extremity radiograph. A full‐length radiograph was taken under fluoroscopy (SONIALVISION G4, SHIMADZU, Tokyo, Japan) in the supine position, one leg at a time, with the knee fully extended and the patella facing forward. The slot radiography technique was utilized to minimize X‐ray distortion and oblique incidence, allowing for an accurate depiction of the entire lower extremity from hip to ankle in a single image.

Full‐length anteroposterior radiographs of the affected lower extremity were acquired within 6 months preoperatively under fluoroscopy using the SONIALVISION G4 system SHIMADZU Corp., Tokyo, Japan). Patients were positioned supine with full knee extension, the patella facing forward, and the ankle in a neutral position (Figure 1). Slot radiography was employed to minimize distortion and parallax errors [20]. Radiographs were analyzed using the NOBORI Picture Archiving and Communication System (PSP Corp., Tokyo, Japan), with angular and linear measurements performed on magnified images of the full tibial length.

Plain radiographs served as the standard imaging modality for preoperative planning in both TKA and HTO procedures, ensuring broad clinical applicability across institutions.

The HKA angle was measured as the angle formed between the line connecting the femoral head to the intercondylar notch and the line extending from the midpoint of the tibial plateau to the center of the talus [17].

The tibia was segmented into three equal regions—proximal, middle, and distal— by drawing horizontal lines at one‐third intervals along its anatomical axis in accordance with the hypothesis that the Japanese tibia has proximal and distal deformities. This classification considered biomechanical principles and intraoperative limitations such as fluoroscopic field of view, which is typically limited to the proximal one‐third of the tibia [2, 6, 9, 15].

**Figure 2:**
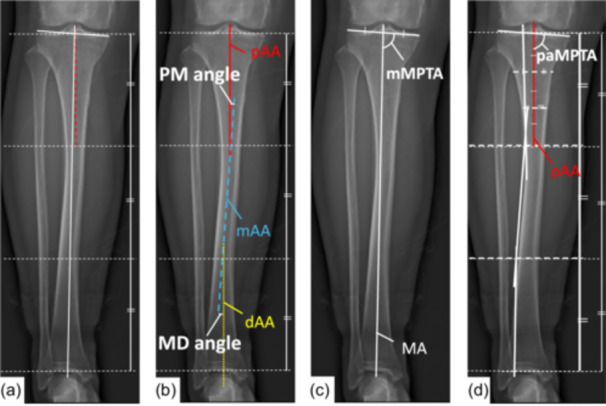
Method for radiographic analysis of tibial alignment. (a) The tibia is divided into three equal segments by horizontal lines. (b) Anatomical axes are defined for each segment: proximal (pAA), middle (mAA), and distal (dAA). Deformity angles are measured between these axes: the proximal‐middle (PM) angle and the middle‐distal (MD) angle. (c) The mechanical medial proximal tibial angle (mMPTA) is measured using the mechanical axis (MA), which connects the center of the tibial plateau to the center of the talus. (d) The proximal anatomical medial proximal tibial angle (paMPTA) is measured using the proximal anatomical axis (pAA).

Within each segment, anatomical axes were defined by connecting the midpoint of the medial–lateral bone diameter across equidistant cross‐sections. These were designated as the proximal anatomical axis (pAA), middle anatomical axis (mAA), and distal anatomical axis (dAA). The proximal–middle (PM) angle was defined as the angle between the pAA and mAA, while the middle–distal (MD) angle was defined as that between the mAA and dAA. Negative values indicated varus alignment, and positive values denoted valgus alignment (Figure 2a,b).

The mechanical axis of the tibia was defined as a line drawn from the midpoint of the tibial plateau to the center of the talus. The mMPTA was measured as the medial angle between the mechanical axis and the tibial articular surface. Conversely, the paMPTA was measured as the angle between the pAA and the tibial articular surface. The difference between these two angles was calculated as ΔMPTA = mMPTA − paMPTA (Figure 2c,d).

Based on these anatomical definitions, a classification system of Japanese tibial morphology was established. For both PM and MD angles, it was defined as follows: varus, <−1°; neutral, −1° to 1°; and valgus, >1°.

This ±1° threshold was selected for its clinical relevance in osteotomy planning, where angular measurements are typically calculated to the first decimal place to determine correction values. This threshold aligns with established clinical practice for distinguishing meaningful deformities from measurement variability. The ±1° cutoff effectively distinguishes normal anatomical variation from deformities that would influence surgical planning and correction strategies.

**Figure 3:**
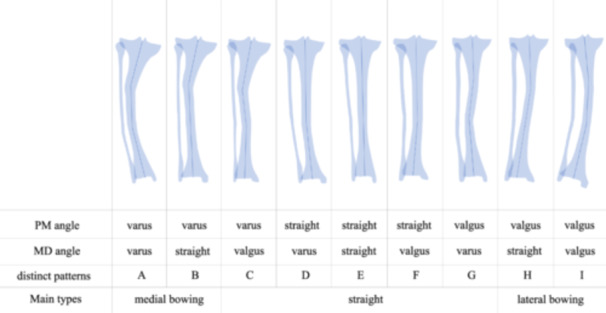
Classification of tibial deformity patterns based on proximal and distal anatomical variations. Nine subgroups (A–I) were identified based on combinations of PM (Proximal‐Middle) and MD (Middle‐Distal) angles. These were consolidated into three main types: varus type (groups A and B), straight type (Groups C–G), and valgus type (Groups H and I). Each illustration represents a schematic view of the tibia, demonstrating characteristic deformity patterns. Varus and valgus deformities are defined as angles less than −1° and greater than 1°, respectively, while straight alignment is defined as angles between −1° and 1°.

Nine unique morphological patterns (Groups A–I) were defined based on combinations of PM and MD angles (Figure 3) and were further grouped into three functional tibial types: medial bowing (Groups A, B), proximal varus deformity without distal compensatory valgus; straight (Groups C–G), either anatomically neutral or exhibiting compensatory alignment between segments; and lateral bowing (Groups H, I), proximal valgus deformity without distal compensation.

### Statistical analysis

Statistical analyses were conducted to evaluate group differences and measurement reliability. Data normality was assessed using the Shapiro–Wilk test. For comparisons among the three tibial types, the Kruskal–Wallis test was used for continuous variables (e.g., age, BMI and BMD), while chi‐square tests were applied to categorical variables (e.g., sex, K–L grade, and surgical procedures).

To compare mMPTA and paMPTA, two‐tailed paired *t* tests were performed after confirming normal distribution. These tests were also conducted within each tibial type subgroup. One‐way analysis of variance was used to compare mMPTA, paMPTA, and ΔMPTA across the three types. Post hoc pairwise comparisons were performed using Scheffé′s test, chosen for its robustness with unequal group sizes (medial bowing: *n* = 49; straight: *n* = 111; lateral bowing: *n* = 33). A significance threshold of *p* < 0.05 was applied to all analyses.

Intraoperative and interobserver measurement reliability was assessed using intraclass correlation coefficients (ICCs). Intraobserver reliability was evaluated on 30 randomly selected knees, with measurements repeated after at least 2 weeks. The ICCs were as follows: intraobserver: mMPTA = 0.987, paMPTA = 0.955, PM angle = 0.902, MD angle = 0.810; interobserver: mMPTA = 0.988, paMPTA = 0.989, PM angle = 0.995, MD angle = 0.990. All ICC values exceeded 0.75, indicating good to excellent reliability.

Statistical analyses were conducted using IBM SPSS Statistics version 28.0 (IBM Japan, Tokyo, Japan).

## RESULTS

A total of 193 knees were included in the study, with TKA performed in 99 knees (51.3%) and HTO in 94 knees (48.7%). The mean patient age was 70.9 ± 10.2 years, with a predominance of females (139 females and 54 males). The average BMI was 25.2 ± 3.7 kg/m², and the mean BMD was 1.2 ± 0.3 g/cm². Radiographic evaluation revealed a mean HKA angle of −5.7° ± 4.0°, with TKA patients showing −7.5° ± 4.4° and HTO patients showing −3.7° ± 2.4°. K–L grading showed a predominance of advanced OA, with Grades III and IV accounting for 65.8% of knees; specifically, Grade I was observed in 12 knees (6.2%), Grade II in 54 knees (28.0%), Grade III in 72 knees (37.3%) and Grade IV in 55 knees (28.5%; Table 1).

**Table 1:** Demographic data of the patients.

	Total (*n* = 193)	Medial bowing (*n* = 49)	Straight (*n* = 111)	Lateral bowing (*n* = 33)	*p* value
Sex (male/female)	54/139	13/36	33/78	8/25	0.799
Age (years)	70.9 ± 10.2	73.5 ± 7.7	69.5 ± 11.1	71.6 ± 9.7	0.150
BMI (kg/m²)	25.2 ± 3.7	24.9 ± 3.3	25.1 ± 3.8	26.1 ± 3.7	0.348
BMD (g/cm²)	1.2 ± 0.3	1.1 ± 0.2	1.2 ± 0.3	1.2 ± 0.3	0.933
HKA (°)	−5.7 ± 4.0	−7.6 ± 5.1	−5.1 ± 3.4	−4.7 ± 3.0	*0.004*
K–L grade (Ⅰ/Ⅱ/Ⅲ/Ⅳ)	12/54/72/55	1/14/19/15	11/33/40/27	0/7/13/13	0.187
Surgical procedure (TKA/HTO)	99/94	26/23	52/59	21/12	0.23

*Note*: Continuous data are reported as ‘mean ± standard deviation.’

Abbreviations: BMI, body mass index; BMD, bone mineral density; HKA, hip‐knee‐ankle angle; HTO, high tibial osteotomyK–L grade, Kellgren–Lawrence osteoarthritis grade; TKA, total knee arthroplasty.

Morphological analysis identified tibial deformities in a substantial proportion of knees, with 131 knees (67.9%) exhibiting proximal deformities and 98 knees (50.8%) showing distal deformities. These deformities were classified into nine proximal–distal alignment patterns (Table 2): three involving proximal varus (Group A: proximal and distal varus, 15 knees [7.8%]; Group B: proximal varus with straight distal, 34 knees [17.6%]; Group C: proximal varus with distal valgus, 21 knees [10.9%]); three with straight proximal alignment (Group D: with distal varus, 10 knees [5.2%]; Group E: straight throughout, 34 knees [17.6%]; Group F: with distal valgus, 18 knees [9.3%]); and three involving proximal valgus (Group G: with distal varus, 28 knees [14.5%]; Group H: with straight distal, 27 knees [14.0%]; Group I: proximal and distal valgus, six knees [3.1%]). These nine groups were further consolidated into three main morphological types: medial bowing type (Groups A and B, 49 knees [25.4%]), straight type (Groups C–G, 111 knees [57.5%]), and lateral bowing type (Groups H and I, 33 knees [17.1%]).

**Table 2:** Distribution of tibial morphological patterns based on proximal and distal anatomical variations.

Main types	Medial bowing		Straight					Lateral bowing		Total
Type total (%)	49 (25.4)		111 (57.5)					33 (17.1)		193
Distinct patterns	A	B	C	D	E	F	G	H	I	
Number of cases (%)	15 (7.8)	34 (17.6)	21 (10.9)	10 (5.2)	34 (17.6)	18 (9.3)	28 (14.5)	27 (14.0)	6(3.1)	

*Note*: Nine morphological patterns (A–I) based on PM and MD angle combinations were consolidated into three main types: medial bowing (A, B), straight (C–G), and lateral bowing (H, I). Values represent the number and percentage of cases in each category.

Abbreviations: MD, middle‐distal angle; PM, proximal‐middle angle.

**Figure 4:**
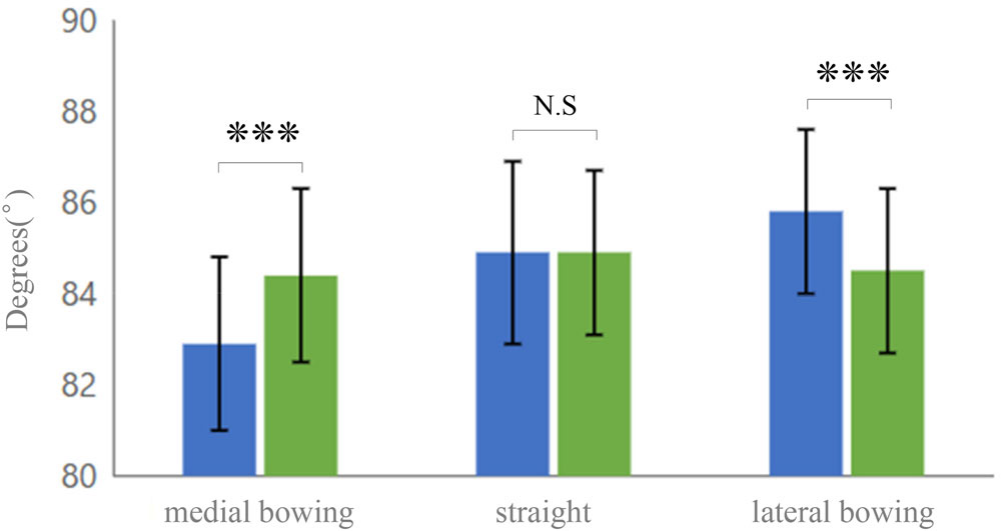
Comparison of mechanical MPTA (mMPTA, blue) and anatomical MPTA (aMPTA, green) values across morphological types. Error bars represent standard deviation. ***p < 0.001. MPTA, medial proximal tibial angle; N.S, not significant.

**Figure 5:**
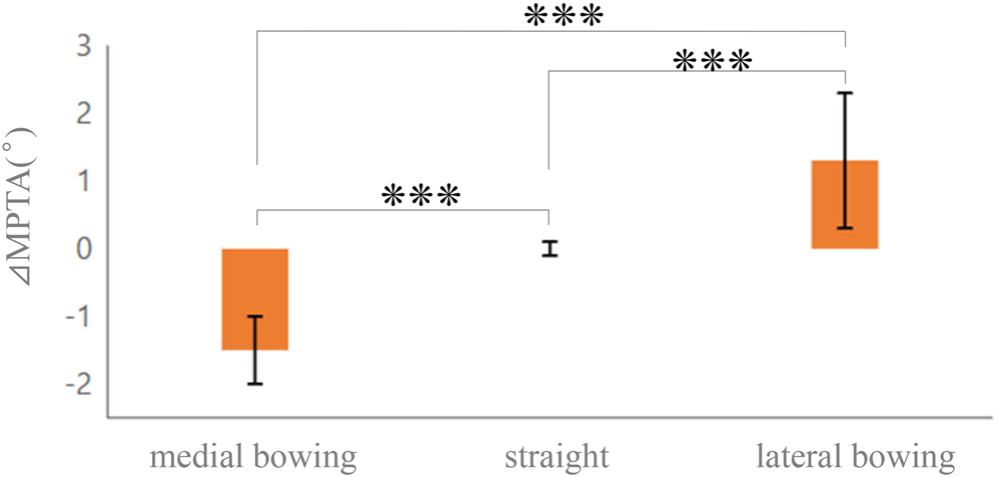
ΔMPTA values by morphological type. Error bars represent standard deviation. ***p < 0.001 for all comparisons. MPTA, medial proximal tibial angle.

Comparative analysis revealed no statistically significant differences in baseline characteristics among the three morphological types, with no significant differences in gender distribution (*p* = 0.799), age (*p* = 0.150), BMI (*p* = 0.348), BMD (*p* = 0.933), or K–L grade distribution (*p* = 0.187), indicating that the observed morphological variations were not attributable to differences in patient demographics or osteoarthritic severity (Table 1). Additionally, chi‐square analysis revealed no significant association between morphological type and surgical procedure *p* = 0.23), indicating that the choice between TKA and HTO was independent of tibial morphology. However, significant differences were observed between mechanical and anatomical measurements among the morphological types (Table 3, Figure 4). In the overall cohort, discrepancies were relatively minor, with a mean mMPTA of 84.5° ± 2.2°, paMPTA of 84.7° ± 1.8°, and ΔMPTA of 0.2° ± 1.2° (*p* = 0.03). In contrast, subgroup analysis revealed distinct patterns: In the medial bowing type, mMPTA was significantly lower (82.9° ± 1.9°) than paMPTA (84.4° ± 1.9°), resulting in a negative discrepancy (ΔMPTA: −1.5° ± 0.5°, range: −2.6° to −0.5°, *p* < 0.001); in the straight type, mMPTA (84.9° ± 2.0°) and paMPTA (84.8° ± 1.8°) were nearly identical, with ΔMPTA of 0.0° ± 0.7°, range: −1.9° to 1.4°, (*p* = 0.60); and in the lateral bowing type, mMPTA was higher (85.8° ± 1.8°) than paMPTA (84.5° ± 1.8°), resulting in a positive discrepancy (ΔMPTA: 1.3° ± 1.0°, range: 0.1° to 3.0°, *p* < 0.001 (Figure 5). Differences between mechanical and anatomical alignment were observed across different morphological types.

**Table 3:** Comparison of mechanical and anatomical MPTA measurements across morphological types.

Type	mMPTA (°)	paMPTA (°)	ΔMPTA (°)	Range (°)	*p* value
Total	84.5 ± 2.2	84.7 ± 1.8	0.2 ± 1.2	−2.6 to 3.0	0.03
Medial bowing	82.9 ± 1.9	84.4 ± 1.9	−1.5 ± 0.5	−2.6 to −0.5	<0.001
Straight	84.9 ± 2.0	84.8 ± 1.8	0.0 ± 0.7	−1.9 to 1.4	0.600
Lateral bowing	85.8 ± 1.8	84.5 ± 1.8	1.3 ± 1.0	0.1 to 3.0	<0.001

*Note*: mMPTA, paMPTA, and their difference (ΔMPTA) for the total cohort and each morphological type. *p*‐values indicate the significance of differences between mMPTA and paMPTA. ΔMPTA = mMPTA − paMPTA. Range indicates minimum to maximum values for ΔMPTA within each group.

Abbreviations: MPTA, medial proximal tibial angle; mMPTA, mechanical medial proximal tibial angle; paMPTA, proximal anatomical medial proximal tibial angle

## DISCUSSION

This study revealed distinct characteristics of Japanese tibial morphology that challenge conventional assumptions. The analysis demonstrated that Japanese tibiae display complex morphological patterns, with a high prevalence of deformities in both the proximal (67.9%) and distal (50.8%) regions. The data dispute the traditional view of equivalence between mMPTA and paMPTA in this population. Such morphological variations provide a practical framework to improve surgical accuracy by emphasizing the importance of considering morphological type‐specific differences in these measurements during correction.

Initially, nine distinct morphological patterns emerged from the analysis of proximal and distal deformity combinations. These were subsequently consolidated into three clinically relevant types—medial bowing (25.4%), straight (57.5%), and lateral bowing (17.1%). This consolidation was based on clinical observations of numerous cases with significant distal tibial deformities, which influenced overall alignment and potentially affected surgical outcomes. This classification system offers a practical guide for surgical planning, allowing surgeons to anticipate compensatory distal deformities that may be overlooked when focusing solely on proximal tibial anatomy during procedures such as HTO. Notably, these morphological variations showed no significant correlation with patient demographics such as age, BMI, BMD or K–L grade, suggesting they represent inherent anatomical traits rather than degenerative changes. The independence between morphological classification and surgical procedure selection (*p* = 0.23) confirms that these observed tibial morphological variations represent inherent anatomical characteristics rather than treatment‐related artifacts. This finding strengthens the validity of this morphological classification system and ensures that the identified ΔMPTA patterns reflect true anatomical differences rather than selection bias toward specific surgical indications.

HKA angles differed significantly among morphological types (*p* = 0.004), consistent with the expectation that local tibial deformities impact overall limb alignment. Previous studies have documented distinct tibial deformities in Asian populations, particularly among Japanese patients [1, 17, 24, 31, 32, 37, 38]. The epidemiology of knee OA reflects these morphological differences, with Asian populations—especially Japanese women—exhibiting higher prevalence rates than Caucasians [8, 29]. Earlier research primarily focused on proximal deformities, reporting varying degrees of proximal tibial angular differences [1, 22, 24]. These findings extend these observations by demonstrating the clinical significance of combined proximal and distal deformities, highlighting the limitations of focusing solely on proximal anatomy. This three‐segment analysis revealed that nearly half of the subjects had distal deformities with a substantial impact on overall alignment.

This radiographic protocol used non‐weight‐bearing supine positioning, which was specifically chosen to optimize tibial morphological assessment. This approach eliminates potential confounding factors such as knee flexion and dynamic tibial distortion that may occur during weight‐bearing, providing optimal conditions for accurate evaluation of constitutional tibial deformities. Additionally, since intraoperative measurements during HTO are performed under non‐weight‐bearing conditions, this preoperative measurement are directly applicable to surgical planning.

Environmental and lifestyle factors may contribute to these morphological traits. The concept of 'constitutional varus', based on Hueter–Volkmann′s law, suggests that mechanical loading during growth influences tibial morphology [6]. Traditional Japanese postures, such as knee‐folded sitting, common among middle‐aged and elderly individuals, may contribute to these deformities. Supporting this, comparative data show a higher prevalence of knee pain among Japanese women living in rural Japan than Japanese–American women in Hawaii (41% vs. 21%; odds ratio, 4.4 after BMI adjustment), despite similar genetic backgrounds [4, 22]. These findings implicate environmental and lifestyle factors in the development of knee pathology, though their direct relationship to tibial deformities remains unclear.

This classification system has important implications for preoperative planning in HTO. Recognizing distal deformities enables more accurate prediction of true mechanical alignment and appropriate surgical correction. This is especially crucial where distal compensatory deformities may mask proximal varus or valgus deformities, potentially resulting in under‐ or over‐correction if unaccounted for. The concept of individualized surgical approaches that account for patient‐specific morphological characteristics has gained increasing recognition in modern knee surgery [3, 12, 14, 16].

A key finding from this MPTA analysis was a systematic discrepancy between mMPTA and paMPTA measurements. This discrepancy was most pronounced in the medial bowing type (25.4%), where anatomical measurements during surgery appeared larger than the actual mechanical alignment (ΔMPTA: −1.5° ± 0.5°, *p* < 0.001). The straight type (57.5%) showed negligible difference (ΔMPTA: 0.0° ± 0.7°), explaining the apparent reliability of traditional approaches. Both medial and lateral bowing types exhibited significant, predictable deviations (lateral bowing type: ΔMPTA: +1.3° ± 1.0°, *p* < 0.001), necessitating reconsideration of conventional measurement methods. While mean discrepancies appear modest, examination of the full range reveals clinically significant individual variations. Schröter et al. reported surgical accuracy in HTO of 1.7°–2.1°, with the important question being how surgical accuracy can be improved [30]. In this cohort, ΔMPTA values ranged from −2.6° to +3.0°, representing a total span of 5.6°. Individual cases in the lateral bowing group showed discrepancies as large as +3.0°, while medial bowing cases reached −2.6°. These substantial individual variations could lead to clinically significant measurement errors if morphological variations are not considered. These non‐straight morphologies together accounted for 42.5% of this cohort, underscoring the widespread nature of this potential measurement error in Japanese patients. The systematic discrepancy between mechanical and anatomical MPTA measurements identified in this study reflects the measurement challenges encountered in actual clinical practice. While there is growing interest in 3D analysis techniques, and Veerman et al. have reported the importance of 3D lower limb alignment analysis [36], the reality is that intraoperative measurements must rely on 2D fluoroscopic imaging with limited field of view. Therefore, improving the accuracy of currently available 2D measurements is clinically crucial.

Given these measurement challenges, these results have immediate clinical relevance, particularly for intraoperative fluoroscopic measurements during HTO. This research differs from previous short‐segment studies by providing practical correction factors for the systematic discrepancy between preoperative planning and intraoperative measurements. The observed differences between mechanical and anatomical MPTA provide an anatomical basis for previously reported measurement uncertainties. Hankemeier et al. demonstrated that techniques such as cable methods and grids with lead‐impregnated reference lines can help achieve the intended intraoperative mechanical axis but only offer momentary evaluation and may be prone to technical errors [13]. Similarly, Gebhard et al. reported that conventional intraoperative leg axis control using cable methods is not always precise [10]. Kubota et al. further identified a significant risk of under‐correction with intraoperative MPTA measurements compared with alignment rod methods [19]. Nakamura et al. proposed using an MPTA measurement film on fluoroscopic monitors for intraoperative confirmation and adjustment during open wedge HTO [25].

This morphological classification and ΔMPTA findings suggest these discrepancies stem from inherent morphological variation (Table 4). The medial bowing type′s significant negative ΔMPTA likely explains systematic under‐correction when morphological differences are not accounted for intraoperatively. Surgeons relying on fluoroscopic anatomical MPTA measurements without morphological adjustment may inadvertently under‐correct varus deformities.

**Table 4:** Summary of tibial morphological types with clinical implications for high tibial osteotomy.

Morphological type	Definition	PM angle	MD angle	Prevalence *n* (%)	Mean ΔMPTA ± SD (°)	Clinical implication
Medial bowing	Proximal varus deformity without distal compensatory valgus	<−1° (varus)	≤1° (varus to neutral)	49 (25.4)	−1.5 ± 0.5	Under‐correction risk if not adjusted
Straight	Anatomically neutral or exhibiting compensatory alignment between segments	Any alignment	Any alignment	111 (57.5)	0.0 ± 0.7	Conventional measurement reliable
Lateral bowing	Proximal valgus deformity without distal compensation	>1° (valgus)	≥−1° (neutral to valgus)	33 (17.1)	+1.3 ± 1.0	Over‐correction risk if not adjusted

*Note*: Three main morphological types with their definitions, prevalence, mean ΔMPTA values, and clinical implications for intraoperative measurement during HTO procedures. ΔMPTA = mMPTA − paMPTA.

Abbreviations: PM, proximal‐middle angle; MD, middle‐distal angle; ΔMPTA, difference between mechanical and proximal anatomical medial proximal tibial angle; SD, standard deviation.

Building on Nakamura et al. fluoroscopic technique, this study proposes an enhanced approach that incorporates morphology‐specific ΔMPTA adjustments. Two practical strategies emerge from these findings. Preoperative identification of morphological type, combined with corresponding intraoperative ΔMPTA corrections, would enable surgeons to achieve more accurate alignment while retaining the practicality of fluoroscopic paMPTA measurement. Alternatively, surgeons may directly measure paMPTA intraoperatively on the fluoroscopic monitor and add the planned correction angle to determine the target paMPTA value, eliminating the need for preoperative morphological classification while still accounting for the systematic discrepancies observed in this study. Although seemingly small, differences of 1°–2° are clinically significant in HTO, where precise angular correction is critical for optimal outcomes. This method leverages morphological classification to improve the precision of intraoperative fluoroscopic assessment.

In approximately 42.5% of Japanese patients with non‐straight tibial morphology, fluoroscopic paMPTA measurements should be adjusted based on morphological type. For optimal surgical precision, preoperative morphological classification and corresponding intraoperative ΔMPTA correction are essential. Implementing this morphology‐adjusted measurement protocol addresses systematic measurement discrepancies while maintaining the practical benefits of fluoroscopic guidance, potentially improving long‐term clinical outcomes through more precise correction.

## LIMITATIONS

This study has several limitations that warrant consideration. First, this cohort included patients undergoing both TKA and HTO, which may over‐represent more severe deformities compared with the general Japanese population with knee OA, and focused exclusively on patients with varus alignment, which may limit the generalizability of these findings to patients with valgus or neutral lower limb alignment. Second, this radiographic protocol did not include measurements required for established international knee phenotype classification systems such as CPAK classification or functional knee phenotypes. This limits direct comparison with international classification systems. Third, this analysis was based solely on frontal‐plane radiographs, which precluded three‐dimensional assessment of tibial torsion and sagittal‐plane deformities achievable via computed tomography. This limitation is notable, as rotational alignment can impact the accuracy of angular measurements derived from two‐dimensional images. Additionally, these radiographs were acquired in the supine position without weight‐bearing, while preoperative planning is typically performed using standing radiographs. The potential impact of this difference on this morphological measurements remain unclear. Fourth, while we employed a standardized method for tibial segmentation, this approach may not fully capture individual variations in deformity patterns. Finally, the retrospective, single‐center design and absence of a non‐Japanese comparison group may limit the generalizability of these findings.

Future research incorporating three‐dimensional imaging, comparative studies across ethnic populations, and prospective validation of this classification system′s clinical impact would strengthen these findings.

## CONCLUSION

This study investigated tibial deformities in Japanese patients with varus knee OA, revealing previously unrecognized complexity in the relationship between mMPTA and paMPTA measurements. This analysis identified distinct patterns of combined proximal and distal deformities, which were categorized into three morphological types. We found significant and systematic differences in ΔMPTA among these groups: the medial bowing type (25.4% of cases) exhibited a mean ΔMPTA of −1.5° ± 0.5°, the straight type (57.5%) showed minimal difference (0.0° ± 0.7°), and the lateral bowing type (17.1%) demonstrated a ΔMPTA of +1.3° ± 1.0°. Incorporating these morphology‐specific adjustments into intraoperative targets, guided by preoperative classification, could enhance the accuracy of HTO and potentially improve clinical outcomes in Japanese patients with knee OA Clinically, this three‐type classification system provides surgeons with a practical tool to identify the 42.5% of patients at risk for measurement discrepancies and apply predictable correction factors during fluoroscopic verification. This approach may reduce systematic under‐ or over‐correction errors and improve long‐term surgical outcomes (Table 4).

## AUTHOR CONTRIBUTIONS

**Etsuko Tabata**: Study design; writing; image acquisition; data collection; data analysis. **Ryuichi Nakamura**: Study design; data analysis; review and editing. **Hiromi Yamaguchi**: Data analysis. **Ryosuke Asa**: data collection. **Masaki Takahashi**: Data curation. **Kazunari Kuroda**: Data curation. **Yasuo Katsuki**: Supervision.

## CONFLICT OF INTEREST STATEMENT

R.N. reports consulting fees from OSferion Biomaterials and AUSPICIOUS outside the submitted work. All other authors declare no conflict of interest.

## ETHICS STATEMENT

The institutional review board of Yawata Medical Center (Approval No. 2021‐11).

## Data Availability

The data that support the findings of this study are available on request from the corresponding author. The data are not publicly available due to privacy or ethical restrictions.
